# DNA methylation in infants with low and high body fatness

**DOI:** 10.1186/s12864-020-07169-7

**Published:** 2020-11-09

**Authors:** Pontus Henriksson, Antonio Lentini, Signe Altmäe, David Brodin, Patrick Müller, Elisabet Forsum, Colm E. Nestor, Marie Löf

**Affiliations:** 1 Department of Health, Medicine and Caring Sciences, Linköping University, 58183 Linköping, Sweden; 2 Crown Princess Victoria Children’s Hospital, and Department of Biomedical and Clinical Sciences (BKV), Linköping University, Linköping, Sweden; 3 Department of Biochemistry and Molecular Biology, Faculty of Sciences, University of Granada, Granada, Spain; 4 Instituto de Investigación Biosanitaria ibs.GRANADA, Granada, Spain; 5 Department of Biosciences and Nutrition, Karolinska Institutet, Huddinge, Sweden; 6 Department of Biomedical and Clinical Sciences (BKV), Linköping University, Linköping, Sweden

## Abstract

**Background:**

Birth weight is determined by the interplay between infant genetics and the intrauterine environment and is associated with several health outcomes in later life. Many studies have reported an association between birth weight and DNA methylation in infants and suggest that altered epigenetics may underlie birthweight-associated health outcomes. However, birth weight is a relatively nonspecific measure of fetal growth and consists of fat mass and fat-free mass which may have different effects on health outcomes which motivates studies of infant body composition and DNA methylation. Here, we combined genome-wide DNA methylation profiling of buccal cells from 47 full-term one-week old infants with accurate measurements of infant fat mass and fat-free mass using air-displacement plethysmography.

**Results:**

No significant association was found between DNA methylation in infant buccal cells and infant body composition. Moreover, no association between infant DNA methylation and parental body composition or indicators of maternal glucose metabolism were found.

**Conclusions:**

Despite accurate measures of body composition, we did not identify any associations between infant body fatness and DNA methylation. These results are consistent with recent studies that generally have identified only weak associations between DNA methylation and birthweight. Although our results should be confirmed by additional larger studies, our findings may suggest that differences in DNA methylation between individuals with low and high body fatness may be established later in childhood.

**Supplementary Information:**

**Supplementary information** accompanies this paper at 10.1186/s12864-020-07169-7.

## Background

Epigenetic variations, such as DNA methylation, have been associated with a growing number of chronic diseases and conditions, including obesity [1–5]. Interestingly, the intrauterine environment may alter DNA methylation patterns in the developing embryo and associations between DNA methylation in neonatal blood and maternal body mass index (BMI), gestational weight gain and smoking have been reported [6, 7]. These associations are supported by animal models in which diet-induced epigenetic changes and their associated phenotypes have been transmitted across multiple generations [8–10] and epidemiological studies have hypothesized that extremes in diet may result in altered disease risk in subsequent generations, possibly via an epigenetic mechanism [11, 12]. Moreover, recent studies have identified an association between neonatal blood DNA methylation and birth weight [13, 14]. As birth weight is predictive of several health outcomes later in life [15, 16], DNA methylation has been proposed as a potential molecular mechanism underlying these associations.

How DNA methylation changes may causally contribute to fatness phenotypes in humans remains unresolved, but a growing body of evidence supports the potential of minor epigenetic changes in early development to cause large changes in body composition [17, 18]. Indeed, we previously showed that subtle epigenetic variation in early mouse development can result in profound changes in littermate body composition [18]. Building on our work in mouse, Pospisilik and colleagues [17] have demonstrated that similar transcriptional variance during early development could result in a stable bi-modal phenotype; obese or not-obese. Importantly, identification of an imprinted gene network as causal in the bi-modal phenotype and it’s recapitulation in a cohort of lean and obese children [17], highlights the potential of epigenetic dysregulation in generation of fat related phenotypes in human [19]. Genomic imprinting as a source of epigenetic-driven alterations in body composition are particularly attractive as DNA methylation is essential for establishing and maintaining genomic imprints and genetic loss of imprinting is typically associated with over-growth and metabolic phenotypes [20].

Birth weight is however a nonspecific measure of fetal growth and consists of fat mass and fat-free mass. This is of importance since fat mass and fat-free mass may have different effects on health outcomes in adulthood [21] as well as during childhood and infancy [22, 23]. Noteworthy, the commonly used surrogate measure for body fatness, BMI, as well as birth weight are poor markers of body fatness in infants [24, 25]. The lack of accurate measurements of infant body composition may underlie the typically weak and often divergent associations between DNA methylation and infant birth weight [13, 14]. Therefore, we hypothesized that an accurate measure of body fatness would provide stronger association with infant DNA methylation. Hence, we measured body fatness accurately using air-displacement plethysmography (ADP) in a cohort of healthy full-term infants [26] and generated base-resolution genome-wide maps of buccal cell DNA methylation from the same infants. The advantage of ADP is that body composition (both fat and fat-free body mass) can be measured accurately in a quick and non-invasive manner [27, 28]. To help identify factors that could influence in utero development and associated DNA methylation patterns we also used ADP to measure body composition in the fathers and mothers in gestational week 32, as well as key measures of glucose metabolism and insulin resistance during pregnancy.

Using our novel approach, we found no association between any measures of body fatness in infants and neonatal buccal cell DNA methylation. Moreover, no association between DNA methylation in infant buccal cells and parental body composition or maternal insulin resistance was identified. Thus, our findings suggest that differences in DNA methylation between individuals with low and high body fatness may be established later in childhood.

## Results

### The DNA methylation profile of buccal cells fails to separate newborns with high or low body fatness

**Fig. 1:**
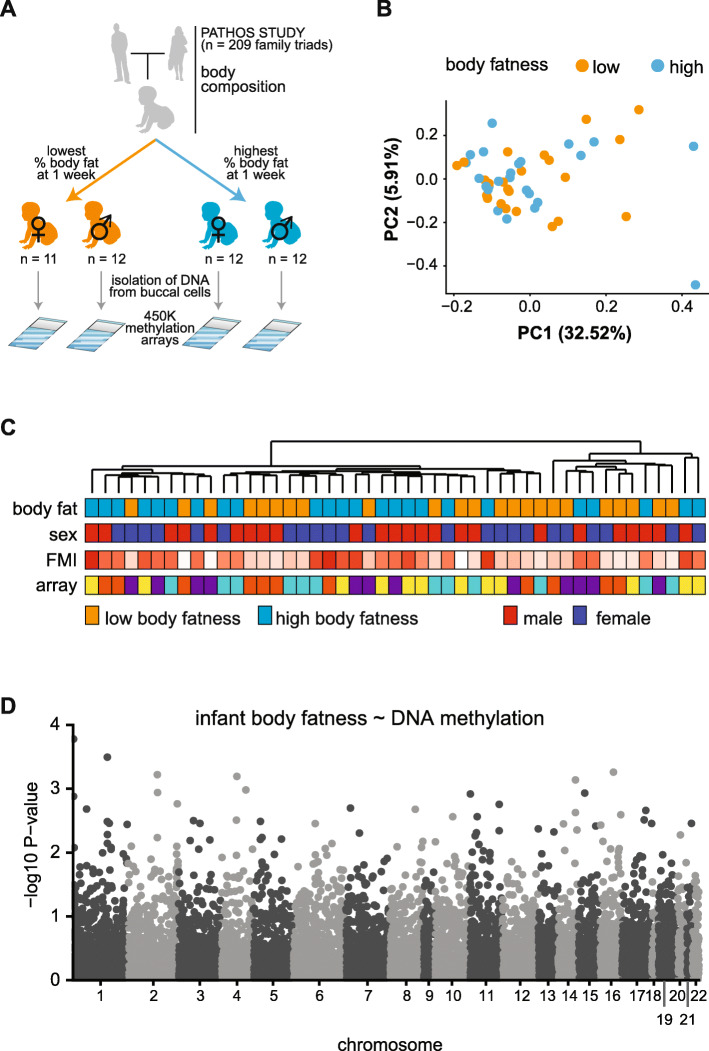
The DNA methylation profile of buccal cells fails to separate newborns with high or low body fatness. (**a**) Outline of experimental design: Buccal cells were isolated from those newborns with the highest (*N* = 23) and lowest (*N* = 24) body fatness enrolled the PATHOS (PArents and THeir OffSpring) study. Genomic DNA was isolated from buccal cells, bisulfite-treated and applied to Illumina® Infinium 450 k DNA methylation arrays. (**b**) Principle components analysis failed to cluster methylation data by fatness (**c**) Unsupervised hierarchical clustering of the same data also failed to separate subjects by body fatness, sex, fat mass index (FMI) or array. (**d**) Manhattan plot showing of association of genome-wide DNA methylation levels with infant body fatness. No probes were significantly associated with body fatness after adjusting for multiple correction (FDR_ADJUSTED_ = 0.05)

In order to study the early programming effects of body fatness in humans we characterized the DNA methylation patterns of buccal cell isolated from those infants identified as having the lowest (*N* = 23) and highest (*N* = 24) body fatness in the PArents and THeir OffSpring (PATHOS) study (Fig. 1a). Body fatness was defined as fat mass (kg) divided by body weight (kg). Characteristics of study participants are summarized in Table 1. No differences in gestational age (independent t-test; *P* = 0.22) or in the fat-free mass index (i.e. fat-free mass normalized for height) (independent t-test; *P* = 0.12) between infants with low and high body fatness were observed. Furthermore, no statistically significant differences (independent t-test) in parental age, parental BMI, parental body composition as well as maternal glycemia were observed between infants with low and high body fatness.

**Table 1:** Characteristic of the infants in the study

	Infants with low body fatness (***n*** = 23)	Infants with high body fatness (***n*** = 24)	
	Value	Value	***P***-value^**a**^
**Infant characteristics**			
Birth weight^b^ (g)	3320 ± 315	4045 ± 416	< 0.001
Gestational age at birth (week)	40.2 ± 1.1	40.6 ± 1.0	0.22
Female sex (n)	11	12	
Male sex (n)	12	12	
Age at measurement (week)	1.1 ± 0.2	1.0 ± 0.3	0.51
Weight at 1 wk. (g)	3271 ± 298	4024 ± 361	< 0.001
Length at 1 wk. (cm)	51.1 ± 1.2	52.4 ± 1.3	0.001
% fat mass at 1 wk.	6.5 ± 2.7	17.6 ± 1.6	< 0.001
BMI at 1 wk. (kg/m^2^)	12.5 ± 0.8	14.6 ± 1.1	< 0.001
Fat mass index at 1 wk. (kg/m^2^)	0.82 ± 0.36	2.57 ± 0.36	< 0.001
Fat-free mass index at 1 wk. (kg/m^2^)	11.7 ± 0.7	12.1 ± 0.8	0.12
**Maternal characteristics**			
Age (year)	30.6 ± 3.3	30.6 ± 4.1	0.98
Pre-pregnancy BMI^2^ (kg/m^2^)	22.6 ± 3.3	22.6 ± 3.1	0.96
BMI^c^ (kg/m^2^)	26.0 ± 3.6	26.7 ± 3.3	0.47
% fat mass^c^	33.8 ± 5.2	33.9 ± 5.2	0.98
Fat mass index^c^ (kg/m^2^)	8.9 ± 2.5	9.2 ± 2.5	0.72
Fat-free mass index^c^ (kg/m^2^)	17.1 ± 1.6	17.5 ± 1.3	0.26
HOMA-IR^c^	1.7 ± 0.7	2.1 ± 0.9	0.74
Glycaemia^c^ (mmol/L)	4.7 ± 0.3	4.8 ± 0.2	0.12
**Paternal characteristics**			
Age (year)	33.9 ± 5.0	32.8 ± 4.4	0.43
BMI^c^ (kg/m^2^)	25.5 ± 4.3	25.3 ± 4.7	0.47
% fat mass^c^	23.7 ± 10.0	23.9 ± 8.8	0.98
Fat mass index^c^ (kg/m^2^)	6.4 ± 3.8	6.3 ± 3.6	0.94
Fat-free mass index^c^ (kg/m^2^)	19.1 ± 1.6	18.9 ± 1.8	0.70

*BMI* Body mass index, *HOMA-IR* homeostasis model assessment-insulin resistance

Values are mean ± SD or n

^a^ Refers to the *P* value of an independent t-test

^b^ Self-reported by the mother

^c^ Measured when mother was in gestational week 32

Genome-wide DNA methylation was determined using llumina® 450 K DNA methylation microarrays. As expected, principal components analysis (PCA) did not cluster the data according to infant body fatness group (Fig. 1b). Unsupervised hierarchical clustering also showed no association with other potential confounders of sex or microarray-based batch effects (Fig. 1c). Thus, no global differences in DNA methylation patterns were observed between infants with high or low body fatness. We next attempted to associate body fatness with DNA methylation levels at individual CpG sites. Despite removal of non-variable sites to increase sensitivity (Supplementary Figure 1B, see methods), no significant associations between DNA methylation levels and infant body fatness (low versus high body fatness) were detected (F-test, *FDR* <  0.05) (Fig. 1d). Thus, differences in infant body fatness were not associated with global or locus-specific changes in DNA methylation in buccal cells.

Given the small sample size of the current study, the absence of association observed may reflect a lack of power to detect smaller yet biologically meaningful associations. Thus, we next sought to determine if those probes showing greatest rank association with infant fatness were enriched for loci identified in previous large-scale EWAS of BMI. No overlap was found between the top 1000 probes reported here and the 156 probes showing statistically significant association between BMI and methylation in the largest EWAS of BMI to date [29]. Furthermore, we found no significant overlap (*N* = 3, *P* > 0.05, Fishers exact test) between the top 1000 probes identified here and those identified in the ALSPAC study of maternal pre-pregnancy BMI and offspring blood DNA methylation (*N* = 1649) [6]. Interestingly, there was also no overlap between the significant probes identified in the ALSPAC (*N* = 1649) study and the EWAS of BMI and adverse outcomes of adiposity (*N* = 156).

### Characterizing the association between parental phenotype and infant DNA methylation

**Fig. 2:**
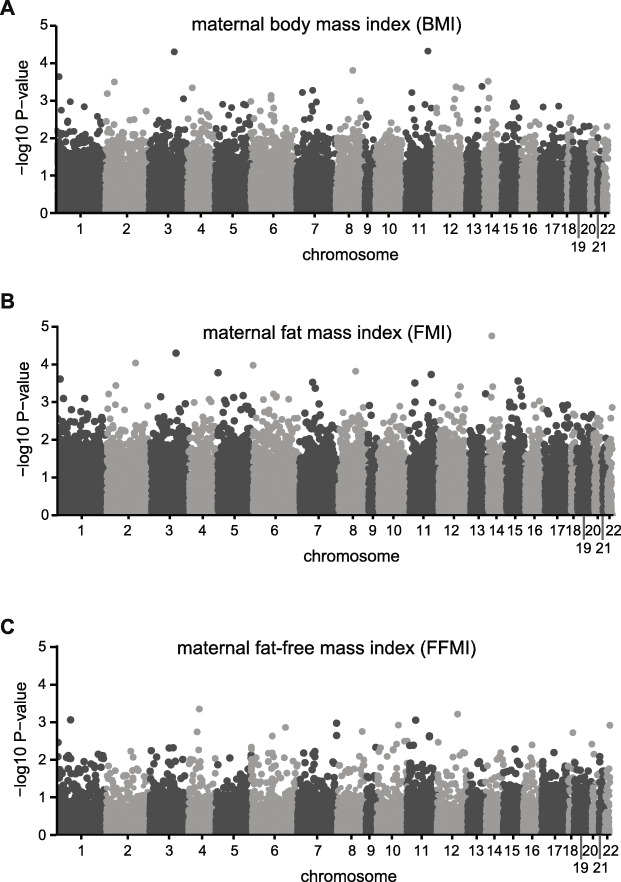
No association between maternal phenotype and infant DNA methylation. (**a-c**) Manhattan plots showing lack of association (FDR_ADJUSTED_ = 0.05) between DNA methylation levels and (**a**) maternal body mass index, (**b**) maternal fat mass index and (**c**) maternal fat-free mass index

The in utero environment has been associated with both infant size (e.g [26, 30, 31]) and methylation levels [6]. Thus, we investigated if the DNA methylation patterns observed in infant buccal cells reflected maternal characteristics during gestation. Again, no loci were significantly associated with maternal BMI, fat mass index or fat-free mass index (Linear regression, *FDR* <  0.05) (Fig. 2a-c). Similarly, no association between maternal insulin resistance and beta-cell function as measured using HOMA-IR) in gestation al week 32 was observed (Supplementary Figure 2A). Finally, there was essentially no evidence for an association of paternal body composition or other infant body composition variables (than body fatness) with infant DNA methylation (Supplementary Figure 2C).

## Discussion

Obesity is a global public health issue and is strongly related to impaired health and quality of life [32]. Birth weight has been related to DNA methylation in infancy [13, 14] as well as health outcomes in later life, such as obesity and mortality [15, 16]. Previous studies have generally reported weak associations between birth weight and DNA methylation in infant blood despite very large sample sizes [13, 14]. However, infants with similar birth weight can have very different levels of body fatness [33]. Consequently, we sought to investigate if accurate assessment of body composition could reveal any biological meaningful associations between DNA methylation and body fatness in newborns.

This study is the first to directly test whether DNA methylation differs between infants with low or high body fatness. Using this novel approach, we did not identify any differences in DNA methylation across the body fatness categories. Moreover, no association between parental body composition and maternal glucose homeostasis with DNA methylation in infants was observed. Whereas our results are somewhat in contrast to previous studies of birth weight and DNA methylation in blood [13, 14], we note that the previously reported differences in DNA methylation associated with birth weight identified are few and typically too small to have a functional impact on gene expression with little overlap between independent studies [14]. Our results may also be compared with previous studies that have examined genetic variation in relation to adiposity in childhood. Although studies have linked a few loci to birth weight these loci have generally not been associated with adiposity later in life [34]. Furthermore, there has been little evidence linking gene variants to infant body fatness [26, 35]. Studies in older children have reported associations between adiposity and several gene variants including single-nucleotide polymorphism in the FTO-gene [36, 37] which is the strongest obesity associated gene variant in adulthood [34]. Interestingly, the influence of FTO gene variant on adiposity appears to be small in infancy but strengthens considerably during childhood [38]. These results may be reconciled with our results and previous studies which have shown association between adiposity and DNA methylation in older children which include studies of both blood [39, 40] and saliva samples [41, 42].

The major strength of the current study is the well-categorized cohort of parents and infants which also included accurate measure of body composition [27, 43]. Nevertheless, the study also has several limitations. First, an important limitation of the study is its relatively small sample size (*N* = 47), which compromises the power of the study to detect small, but significant associations between DNA methylation and body fatness. Calculating significant difference in EWAS using DNA methylation arrays is complex and no standard significance thresholds exist. Using a recently described simulation-based approach to estimating the 5% family-wise error rate for methylation array studies, the current study has 80% predicted power to detect > 5% methylation differences at 25% of CpG sites on the array and > 2% methylation differences at just 4% of sites [44]. Consequently, the current study is limited to detection of relatively large differences in DNA methylation between infant with low and high body fatness. Second, although the study included infants with a wide range in body fatness, they all came from a well-nourished population which motivates further studies in populations with a more heterogeneous nutritional status. Third, the use of buccal epithelial cell (BEC) DNA obtained from buccal swabs, instead of other relevant tissues such as adipose or liver tissue, is a potential limitation of the study. Our use of BECs as a surrogate tissue was motivated by several factors including the relativity easy and non-invasive collection of DNA [45] which was also supported by the fact that we were able to collect DNA from all 209 infants in the PATHOS study [26]. Moreover, a growing body of evidence supports the use of BECs over blood in EWAS [46–48]. DNA methylation patterns in blood are profoundly different from those in most other somatic tissues questioning their choice as a surrogate for non-blood related phenotypes [46, 48]. BECs exhibit higher, and more consistent inter-individual DNA methylation variation, increasing the effective power of BEC EWAS over blood EWAS. In addition, biological and technical replicates of BECs show more stability between samples than blood, reducing noise [49]. Finally, differentially methylated regions (DMRs) in BECs more often overlap known disease-associated SNPs than blood DMRs [47]. Although buccal swabs were carefully performed, BEC preparations can be contaminated with non-epithelial cells, such as lymphocytes, which may lower the predictive power of the study, but would not be expected to vary between groups.

## Conclusion

In conclusion, this study reports no difference in genome-wide DNA methylation between 1-week-old infants with low and high body fatness. Although our findings require confirmation by future larger studies, our results indicate that potential differences in DNA methylation between lean and obese individuals may develop later in childhood.

## Methods

### Study participants

This pilot study utilized data from a previous study called the PATHOS (PArents and THeir OffSpring) study, which investigated associations of parental and infant body composition early in life [26, 50]. In order to maximize the statistical power and since we hypothesized that the largest differences in DNA methylation would be between extremes in infant body fatness, we selected the 24 infants (12 girls and 12 boys) with the lowest body fatness (range: 0.9–9.8% fat mass) and the 24 infants (12 girls and 12 boys) with the highest body fatness (range: 15.1–21.1% fat mass) at 1 week of age for this study.

The study was approved by the Regional Ethics Committee (reference numbers: M187–07 and 2012/440–32), Linköping and informed consent, witnessed and formally documented, was obtained from the parents.

### Body composition of infants and their parents

At 1 week of age, infant length and weight were assessed using standardized procedures and subsequently, the body composition of the infants was measuring using ADP and the Fomon model (Pea Pod, COSMED USA, Inc., Concord, CA, USA), see our previous study for more detailed information [50]. The height, weight and body composition of both mothers and fathers were measured after an overnight fast when the mother was in gestational week 32. Briefly, body composition was assess by ADP (Bod Pod, COSMED USA, Inc., Concord, CA, USA) as previously described [30, 50]. Furthermore, a fasting blood sample was collected from the mother to determine plasma glucose and serum insulin. None of the mothers were diagnosed with gestational diabetes. Maternal HOMA-IR (homeostasis model assessment-insulin resistance) was calculated according to Matthews et al. [51]. Maternal pre-pregnancy weight was self-reported at the measurement in gestational week 32. BMI [weight (kg)/height^2^ (m)], fat mass index [FMI; fat mass (kg)/height^2^ (m)], and fat-free mass index [FFMI; fat-free mass (kg)/height^2^ (m)] were calculated.

### DNA extraction and DNA methylation analysis

A buccal swab was performed on the infants and subsequently DNA was extracted using QuickExtract DNA Extraction Solution 1.0 (Epicentre Biotechnologies, Madison, WI, USA). DNA quality was assessed by using Agilent Genomic DNA ScreenTape System and DNA concentration was measured using Qubit fluorometer. DNA was stored in – 20 °C for further analysis. 500 ng of genomic DNA was bisulfate converted with EZ-96 DNA Methylation kit (Zymo Research, Irvine, CA, USA) and genome wide DNA methylation analysis was performed using the Infinium Human Methylation 450 K BeadChip (Illumina, San Diego, CA, USA) according to the manufacturer’s instructions.

### Methylation data analysis

Illumina 450 K DNA methylation data was pre-processed using functional normalization [52] as implemented in minfi [53]. One sample did not pass quality control and was excluded from further analyses (Supplementary Figure 1A). Next, problematic probes were excluded based on general purpose masking [54] and only autosomes were kept. Differentially methylated region (DMR) analysis using the minfi bumphunter function (cutoff = 0.2 and bootstraps (B) = 1000) [53] yielded only two DMRs consisting of single probes with FWERs > 0.05 (data not shown), therefore we focused on single CpGs instead. Differentially methylated probes (DMPs) were identified using the minfi dmpFinder function using default settings [53]. Briefly, continuous variables (BMI, FMI, FFMI and log HOMA-IR) were tested with linear regression and categorical variables (infant body fatness group) were tested with F-tests. To increase statistical power in identifying DMPs after multiple-testing correction, invariant probes related to cell type were filtered as previously described [55] but with a more stringent requirement of at least 20% variability between the 10th and 90th percentile, this represented approximately the top 5th percentile of variable CpGs. The remaining 20,027 filtered DMP *P*-values were false discovery rate (FDR)-corrected to account for multiple testing.

## Supplementary Information

**Additional file 1: Supplementary Figure 1.** DNA methylation quality control and pre-processing. (A) Density plot showing expected bi-modal distribution of DNA methylation values in all samples except one (dashed line) (top) and fraction of failed probe positions per sample based on detection *P*-values (*P* > 0.01) (bottom). (B) Plot showing the variability cutoff below which probes were excluded from linear regression studies of association (see methods). **Supplementary Figure 2.** No association between parental phenotype and infant DNA methylation levels. (A) Manhattan plot showing lack of association (FDR_ADJUSTED_ = 0.05) between infant DNA methylation levels and maternal homeostatic model assessment insulin resistance (HOMA-IR) score. (B) Manhattan plots showing lack of association (FDR_ADJUSTED_ = 0.05) between DNA methylation levels and infant body mass index, infant fat mass index and infant fat-free mass index. (C) Manhattan plots showing lack of association (FDR_ADJUSTED_ = 0.05) between DNA methylation levels and paternal body mass index, paternal fat mass index and paternal fat-free mass index.

## Data Availability

The raw data has been deposited in the public functional genomics data repository, ArrayExpress (https://www.ebi.ac.uk/arrayexpress/), under the accession number E-MTAB-9596. The raw data will be made publicly available upon publication of the article.

## Abbreviations

ADP: Air-displacement plethysmography
BMI: Body mass index
DMP: Differentially methylated probes
FDR: False discovery rate
FFMI: Fat-free mass index
FMI: Fat mass index
HOMA-IR: Homeostasis model assessment-insulin resistance
PATHOS study: PArents and THeir OffSpring study
PCA: Principal components analysis
