# Supplementary figures and images for: DNA methylation in infants with low and high body fatness
Source: BMC Genomics. 2020 Nov 9;21:769. doi: 10.1186/s12864-020-07169-7 (PMC7654595; doi:10.1186/s12864-020-07169-7)

## Supplementary Figure 1

**A**

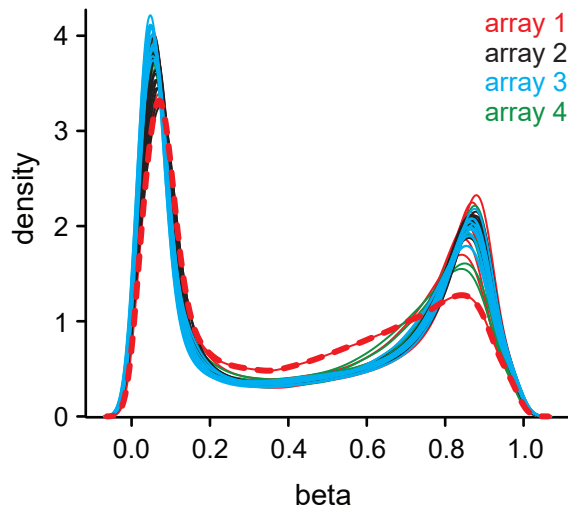

**B**

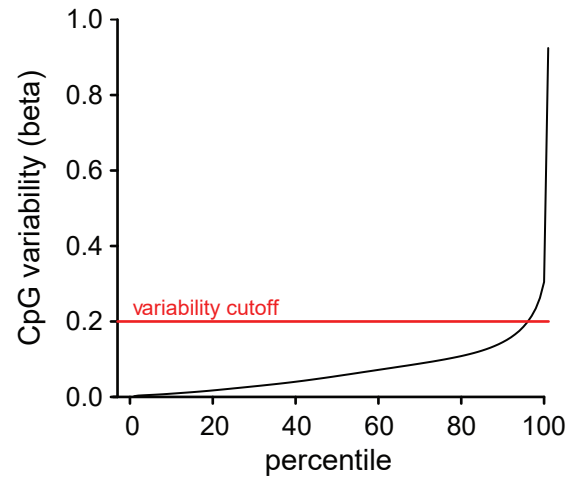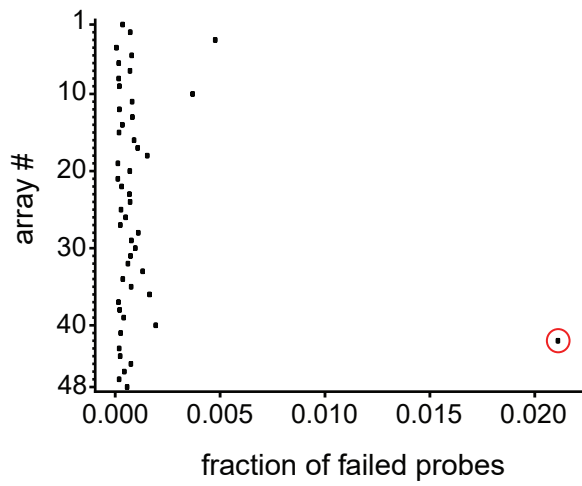

## Supplementary Figure 2

**A**

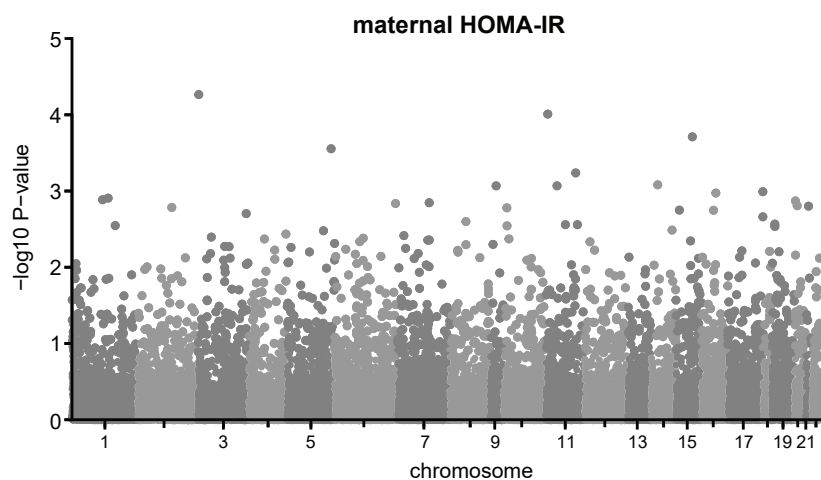

**B**

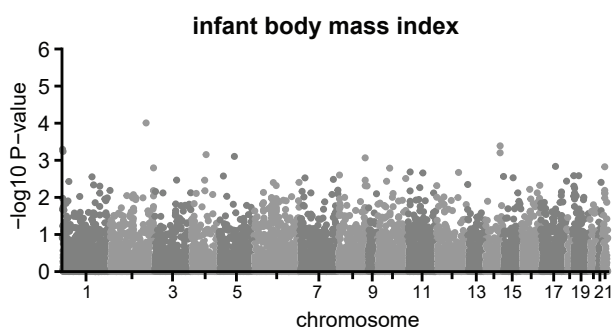

**C**

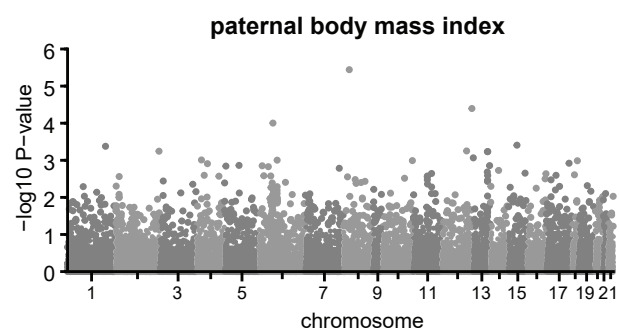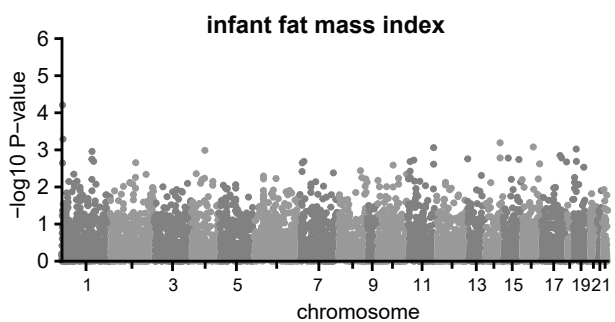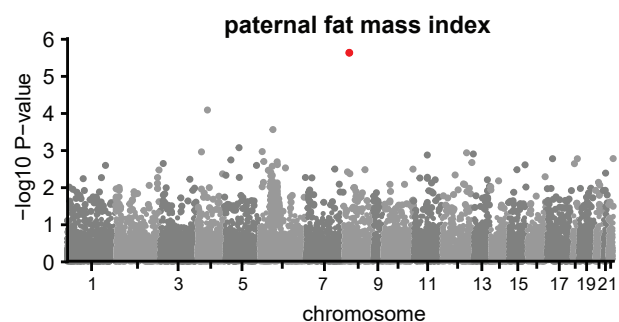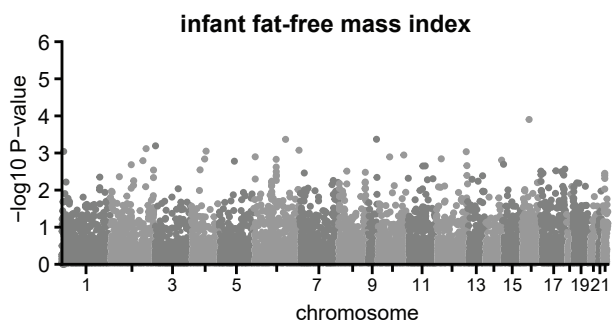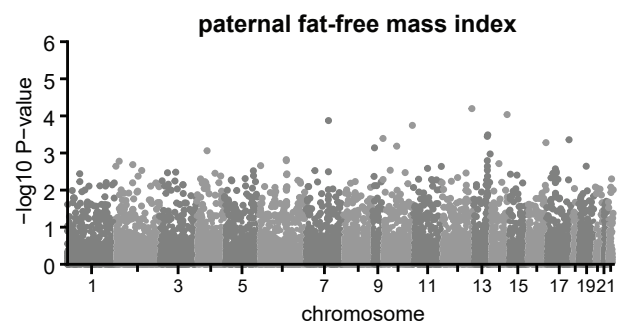

Supplement: Supplementary file 1 — Additional file 1: Supplementary Figure 1. DNA methylation quality control and pre-processing. (A) Density plot showing expected bi-modal distribution of DNA methylation values in all samples except one (dashed line) (top) and fraction of failed probe positions per sample based on detection P-values (P > 0.01) (bottom). (B) Plot showing the variability cutoff below which probes were excluded from linear regression studies of association (see methods). Supplementary Figure 2. No association between parental phenotype and infant DNA methylation levels. (A) Manhattan plot showing lack of association (FDRADJUSTED = 0.05) between infant DNA methylation levels and maternal homeostatic model assessment insulin resistance (HOMA-IR) score. (B) Manhattan plots showing lack of association (FDRADJUSTED = 0.05) between DNA methylation levels and infant body mass index, infant fat mass index and infant fat-free mass index. (C) Manhattan plots showing lack of association (FDRADJUSTED = 0.05) between DNA methylation levels and paternal body mass index, paternal fat mass index and paternal fat-free mass index. [file 12864_2020_7169_MOESM1_ESM.pdf]
